# Effects of Sleep Deprivation and Hazard Types on the Visual Search Patterns and Hazard Response Times of Taxi Drivers

**DOI:** 10.3390/bs13121005

**Published:** 2023-12-08

**Authors:** Long Sun, Meiqi Zhang, Yuanbo Qiu, Changlu Zhang

**Affiliations:** School of Psychology, Liaoning Normal University, Dalian 116029, China; asd3145450056@163.com (M.Z.); aboabo2010@163.com (Y.Q.); zhll66@126.com (C.Z.)

**Keywords:** hazard perception, sleep deprivation, hazard type, visual search pattern, taxi drivers

## Abstract

The present study attempted to explore the effects of sleep deprivation on the visual search patterns and hazard response times of taxi drivers when they encountered different types of hazards. A two (driver groups: sleep deprivation or control) × two (hazard types: covert hazard or overt hazard) mixed experimental design was employed. A total of 60 drivers were recruited, half of whom were in the sleep-deprived group and half of whom were in the control group. A validated video-based hazard perception test that either contained covert hazards (12 video clips) or overt hazards (12 video clips) filmed from the drivers’ perspective was presented to participants. Participants were instructed to click the left mouse button quickly once they detected a potentially dangerous situation that could lead to an accident. Participants’ response time and eye movements relative to the hazards were recorded. The sleep-deprived group had a significantly longer response time and took a longer time to first fixate on covert hazards than the control group, while they had a shorter response time to overt hazards than the control group. The first fixation duration of sleep-deprived drivers was longer than that of the control group for overt hazards, while the duration of the first fixation of the two driver groups was similar for covert hazards. Sleep deprivation affects the visual search patterns and response times to hazards, and the adverse effects of sleep deprivation were worse in relation to covert hazards. The findings have some implications for classifying and evaluating high-risk taxi drivers whose hazard perception ability might be affected by insufficient sleep.

## 1. Introduction

Statistics show that in 2021, there were 1,391,300 taxis in China and approximately 2.7 million taxi drivers [1]. Taxi drivers are one of the major road users accounting for road traffic fatalities [2]. Due to the standards and safety requirements of the work, taxi drivers suffer from high driving exposure and high emotional stress [3]. Such driving characteristics make taxi drivers more susceptible to a lack of sleep, which leads to more dangerous driving behaviors and road accidents [4,5,6].

Numerous studies found that sleep deprivation and poor sleep quality among drivers lead to increased accident risk [4,7,8,9]. Sleep deprivation refers to the deprivation of being unable to meet normal sleep requirements due to personal or work reasons [4,10,11]. Under sleep deprivation conditions, variation in the drivers’ lateral position in a lane increases [12], and the frequency of lane departures [11,13] and velocity variability increase [14]. Insufficient sleep or sleep deprivation affects response times [15] but with mixed results. Bartrim et al. [7] found that sleep restriction had no significant impact on choice response time (CRT) performance, while Saxby et al. [16] found that sleep-deprived drivers have slower braking and steering response times in passive driving. Regarding the effect of sleep deprivation on eye movement, numerous studies have found that sleep-deprived drivers have longer blink times [11,13,17,18], smaller pupil diameters [17], and slower eye movements [13] and saccade amplitude [11] than non-sleep-deprived drivers.

Some studies have found that sleep-deprived drivers have a poor ability to identify and detect hazards [19,20]. Hazard perception, one of the few driving abilities associated with crash risk, is defined as the driver’s ability to detect potentially hazardous events on the road [21]. Typically, hazard perception is measured based on the reaction time paradigm using still images, simulated driving situations, road tests, and clips of normal driving. Prior studies found that response time in hazard perception tests or tasks can distinguish not only novice drivers and experienced drivers [21,22,23,24] but also high-risk drivers and low-risk drivers [25,26,27]. Studies have found that less-experienced drivers respond slower to hazards than experienced drivers (e.g., see [28] for a review), although there are some exceptions [29]. These inconsistent results may be due to the different types of hazards used in the test materials.

Although no experience-related differences were found in the overall response time, Sagberg and Bjørnskau [29] did find some clips that can distinguish novice and experienced drivers. Crundall et al. [22] revealed that some hazard types are indeed more attractive to drivers and sensitive to driving experience. This study examined drivers’ response times and visual search patterns in relation to different hazard types (environmental prediction hazards, behavioral prediction hazards, and dividing and focusing hazards) and found that learners spotted the same number of behavioral prediction hazards but tended to miss more environmental prediction hazards than experienced drivers and driving instructors. Vlakveld [24] classified hazards in the hazard perception task into covert and overt hazards. Covert hazards were those with interrupted visibility, while overt hazards were those with continuous visibility. Specifically, covert hazards were partially or totally blocked in the process of materialization and became visible at the very moment when a maneuver was needed to avoid a collision; overt hazards were totally visible in the process of materialization in front of the camera car. Crundall [30] found that novice drivers were significantly poorer at predicting covert hazards, while experienced drivers were significantly more accurate at identifying all hazard types. Other studies have found that drivers take longer to respond to covert hazards than to overt hazards regardless of their driving experience [23,24]. When driving sleep-deprived, drivers’ actual situational awareness is reduced, which affects driving performance (increasing collisions) [9,31]. Studies have found that covert hazards are difficult to detect due to their interrupted visibility [22,24] but a high level of situational awareness might help drivers detect them quickly. Although taxi drivers can obtain a higher level of situational awareness due to increased driving exposure, it is still unknown whether drivers who suffer from sleep deprivation can detect covert hazards in time to avoid potential collisions.

To the best of our knowledge, only two studies have examined the effects of sleep quality on hazard perception. Smith et al. [19] investigated the difference in hazard perception between high and low sleepiness for novice drivers and experienced drivers, and they found that novice drivers responded significantly slower to hazards, while experienced drivers were relatively unaffected under the condition of low sleepiness. Watling et al. [20] recruited 32 young adults aged between 20 and 25 years to complete a 60-min hazard perception task (including three distinct 20-min time segments to determine time-on-task effects)—when alert and when sleep-restricted—to examine the effect of sleepiness and time-on-task on hazard perception performance and visual scanning behaviors. They found that hazard perception performance decreased and that both the horizontal and vertical eye scanning ranges became restricted across the 60-min session in the alert condition but with a more pronounced effect when sleep-restricted. Although the two studies examined the effects of sleep deprivation on response time and eye movement, further research is needed to determine whether the effects can be found for different hazard types.

The present study aimed to investigate the effects of sleep deprivation and hazard types on the hazard response time and visual search patterns of taxi drivers. Participants were divided into two driver groups (a sleep-deprived group and control group) and were asked to click the left mouse button quickly once they detected a potential road hazard. The hypotheses of the present study are as follows: (1) Previous studies have found that drivers respond slower to covert hazards than to overt hazards [30], and sleep-deprived drivers perform worse with reacting to hazards than normal drivers [20]. Therefore, we expected that compared with the control group, sleep-deprived drivers would respond slower to hazards, especially to covert hazards; (2) Previous studies have found that sleep-deprived drivers have longer blink times, smaller pupil diameters, and smaller saccade amplitude [17,18], we predicted that sleep-deprived drivers may take more time to see the hazard (time to first fixation), and fixate on it longer to process it (total fixation duration) than the control group.

## 2. Methods

### 2.1. Participants

The data were collected using a convenience sample. The present study recruited a total of 100 taxi drivers from Dalian City through traffic broadcasts. Upon knowing the purpose of the study, 90 drivers agreed to participate in this study. All participants had to wear the Actiwatch-GT3X-BT, to monitor their sleep situation, and they also filled out a questionnaire regarding their medicine/drug use history. Twenty-six drivers were excluded either due to medicine use or abnormal sleep patterns. The remaining 64 drivers were asked not to use alcohol, caffeine, or other stimulants that affect cognition or mood for seven days before participating in the experiment. Finally, 60 taxi drivers agreed to participate in the formal experiment and were randomly divided into two groups.

Thirty drivers (eight females) were in the sleep-deprived group, and their ages ranged from 22 to 55 years old, with driving experience between 3 and 30 years. Fourteen drivers in this group had traffic violations, and eleven drivers had traffic accidents in the past two years. Regarding driving frequency, participants drove at least 1~2 days a week, and 73% of the participants drove 5~6 days a week. The remaining 30 drivers (5 females) were in the control group. Their ages ranged from 22 to 55 years old, with driving experience between 3 and 35 years. Seven drivers had traffic violations, and five drivers had traffic accidents in the past two years. Regarding driving frequency, participants drove at least 1~2 days a week, and 67% of the participants drove 5~6 days a week. Detailed demographic information for the two groups is shown in Table 1.

The chi-square test showed that there was no significant difference in the sex ratio between the two driver groups (*χ*^2^ = 0.88, *p* > 0.05). Independent sample t-tests showed that the two driver groups were not significantly different in their age, *t*(58) = 1.00, *p* > 0.05; driving experience, *t*(58) = 0.45, *p* > 0.05; driving frequency, *t*(58) = −0.02, *p* > 0.05; or traffic accidents, *t*(58) = 1.77, *p* > 0.05. The two driver groups were significantly different in the number of traffic violations, *t*(58) = 2.51, *p* = 0.015.

### 2.2. Apparatus and Stimuli

#### 2.2.1. Hazard Perception Test

**Figure 1:**
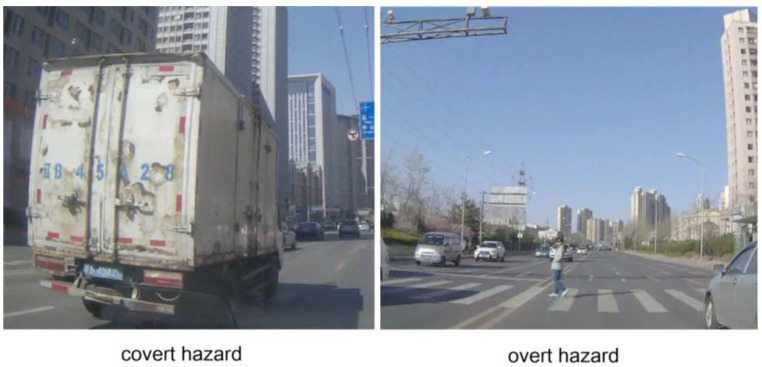
Covert hazard (a large white van occludes a pedestrian walking across the road from the left side); overt hazard (a pedestrian standing in the middle of the road in front of the camera car, walking across the road).

The test contained 28 video clips that were derived from a valid hazard perception test [23]. All of the video clips were filmed from the driver’s perspective in sunny weather conditions. Each clip included only one hazard to obtain an unambiguous response, and the hazard’s onset time and location were random from one clip to another. The video clips in this study have been validated in previous studies, the test score could effectively discriminate experienced drivers from novice drivers [23], and violation-involved taxi drivers from violation-free taxi drivers [27]. Twenty-four video clips were used for formal experiments, and four clips were used for practice. Twelve video clips contained covert hazards, while another twelve video clips contained overt hazards (see Figure 1). The duration of each video clip was within 8~15 s (*M* = 10.13), and the average duration of the two hazard types was not significantly different (*t* = 0.96, *p* > 0.05). Detailed hazard description is shown in Appendix A.

A hazard window was defined for the hazard in each video clip. The hazard window began at the earliest point in time in which the hazard was emerging and ended at the point where a braking or avoidance response by the driver would no longer prevent a collision [29,32]. A traffic police officer and a traffic safety expert were invited to evaluate the beginning and end of the hazard window independently, and the inter-rater reliability ranged from 0.80 to 0.95. In this study, a driver’s response falling outside the hazard window was invalid. The response rate of the participants was above 90% for each clip. In case some participants did not respond to the hazard in some clips, the vacancy value was replaced by the maximum value of the hazard window [33].

#### 2.2.2. The Sleep Quality Scale (SQS)

The Sleep Quality Scale (SQS) is a self-report measure of an individual’s current level of sleep quality. The Chinese version of the SQS has twenty-three items and includes four dimensions: difficulty getting up, difficulty falling asleep, sleep recovery, and daytime dysfunction [34]. The items were rated on a 4-point Likert scale, ranging from 1 (rarely) to 4 (almost always). In this study, total average scores were calculated, with higher scores indicative of worse sleep quality. The internal consistency reliability for the whole scale was 0.89 in this study.

#### 2.2.3. The Stanford Sleepiness Scale (SSS)

The SSS is used to assess the condition of sleepiness tendency [35] and was proven valid among Chinese participants [15]. Participants were asked to complete the SSS by choosing one of seven statements that best described their current state of alertness. The items were rated on a 7-point Likert scale, ranging from 1 (full of energy) to 7 (falling asleep). A higher score indicated a higher tendency toward sleepiness. Rating a score greater than 3 (including 3) represents that the driver is under fatigue status [15].

#### 2.2.4. The Gazepoint GP3 HD Eye Tracker

Visual search behavior was continuously monitored and recorded via the Gazepoint GP3 HD eye tracker, with a 150 Hz sampling rate, a 0.1-degree spatial resolution, and a 0.5~1 degree accuracy. The eye-tracker was around 65 cm away from the participant and 40 cm below their eye level. No equipment was used to restrict participant head movement and they could move their head naturally throughout the study. Calibration utilized a 5-point calibration procedure by Gazepoint Control. Eye-tracking data were imported into Gazepoint Analysis, where relevant indicators were calculated and extracted for analysis. According to the dispersion algorithm, the minimum dispersion considered a fixation is 1 visual degree and the minimum fixation duration is 100 ms.

**Figure 2:**
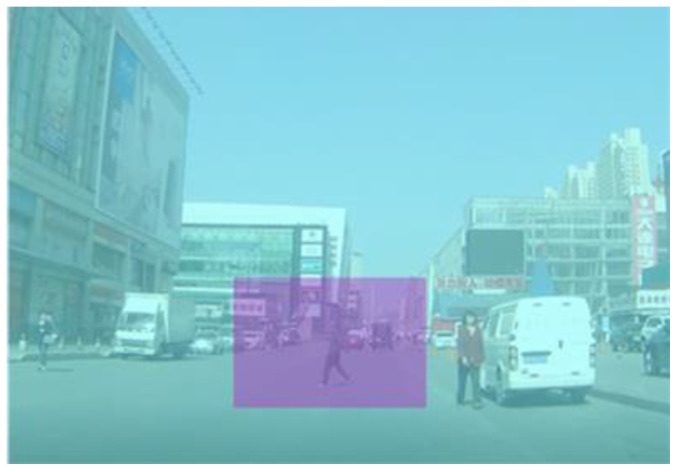
Areas of interest. Note: the purple area is the hazardous zone, and the blue area is the non-hazardous zone.

The dynamic areas of interest (AOIs) were defined for each video clip (see Figure 2). The size of the hazardous zone is 360 × 260 pixels. The AOI covers the hazard’s trajectory from the time of hazard onset to the time the hazard appears in front of the camera car. For covert hazards, the AOI covers the area where a hidden road user first becomes visible rather than the objects that block the road user.

#### 2.2.5. The Actiwatch-GT3X-BT

The Actiwatch-GT3X-BT, a wearable device that measures and records body movements related to daily activities and sleep conditions, was used to record the sleep conditions of participants. Additionally, ActiLife was used to set recording times and export data.

### 2.3. Experimental Design

A 2 × 2 mixed experimental design was employed. The between-group factor was driver group (sleep deprivation or control) and the within-group factor was hazard type (covert hazard or overt hazard). Based on previous research [4,10] on sleep deprivation for the purpose of the present study, sleep deprivation was defined as sleep restriction for at least 24 h (≥24 h awake), and drivers in the control group maintained a normal daily amount of sleep.

The present study included four dependent variables: (1) Response time refers to the time from when participants first fixated on the hazard to the moment when they responded to it; (2) Time to first fixation refers to the time from the onset of the hazard to the moment when participants first fixated on the hazard, representing how fast drivers can detect a hazard; (3) First fixation duration refers to the duration of the first fixation on the hazard; (4) Total fixation duration refers to the amount of attention devoted to the hazard after participants first fixated on it.

### 2.4. Procedure

The entire experiment was conducted in the laboratory of Liaoning Normal University. In the week before the experiment, all participants were asked to wear the Actiwatch-GT3X-BT continuously for 7 days to record their sleep situation. After providing a detailed explanation of the study procedure, all participants completed a demographic questionnaire and filled out the SQS on the first day. Then, all participants were asked to have normal sleep 6 days before the experiment. On the last day, participants assigned to the sleep deprivation group were asked to attend the experiment the next morning at 7 a.m. after a night of sleep deprivation (≥24 h awake) in the lab, while participants assigned to the control group were asked to attend after a night of normal sleep. After a night of sleep deprivation, participants were asked to complete the SQS and the SSS prior to beginning the experiment, and Actiwatch-GT3X-BT data were also checked. Participants could withdraw at any time they want.

The test was displayed on a 17-inch Lenovo computer monitor. The instructions were displayed on the screen, and participants were asked to watch the video clips and to click the left mouse button quickly once they detected a potentially dangerous situation that could lead to an accident.

Participants first practiced with 4 clips to ensure that they understood the instructions accurately. Then, 24 video clips were randomly presented on the screen. The experiment lasted approximately 15 min. Participants were paid CNY 200 for their participation. Sleep-deprived taxi drivers were asked to take a day off after the experiment and were paid an extra CNY 100.

### 2.5. Data Analysis

Data were analyzed using SPSS 18.0. The means and standard deviations of response time, time to first fixation, first fixation duration, and total fixation duration of the sleep-deprived group and the control group were calculated for the covert hazards and overt hazards. Then, repeated-measures ANOVA was conducted on the four dependent variables. The assumptions for mixed ANOVA were met for all dependent variables except for total fixation duration, which was analyzed by applying ‘robust mixed ANOVA’ using R language.

## 3. Results

### 3.1. Questionnaire Results

The results of the independent-sample t-test showed that no significant difference in the SQS scores was found on the first day between the sleep-deprived group (*M* = 2.54, *SD* = 0.55) and control group (*M* = 2.52, *SD* = 0.57), *t*(58) = 0.11, *p* > 0.05. There were significant differences in the SQS scores on the seventh day between the sleep-deprived group (*M* = 3.00, *SD* = 0.35) and control group (*M* = 2.10, *SD* = 0.50), *t*(58) = 8.09, *p* < 0.001. There were significant differences in the SSS scores between the sleep-deprived group (*M* = 4.47, *SD* = 1.67) and control group (*M* = 2.43, *SD* = 1.30), *t*(58) = 5.24, *p* < 0.001.

### 3.2. Response Time and Eye Movement Indicators

The means and standard deviations for all measures are summarized in Table 2. Unless otherwise stated, a two (driver group: sleep deprivation or control) × two (hazard type: covert hazard or overt hazard) repeated-measures ANOVA was run for all measures. The significance level was set at 0.05.

#### 3.2.1. Response Time

For the response time, a significant main effect of hazard type was found, *F*(1, 58) = 683.08, *p* < 0.001, *ηp*^2^ = 0.922, indicating that taxi drivers took longer to respond to covert hazards than overt hazards. A significant main effect of the driver group was also found, *F*(1, 58) = 4.13, *p* < 0.05, *ηp*^2^ = 0.067, indicating that sleep-deprived taxi drivers took significantly longer to react than the control group.

**Figure 3:**
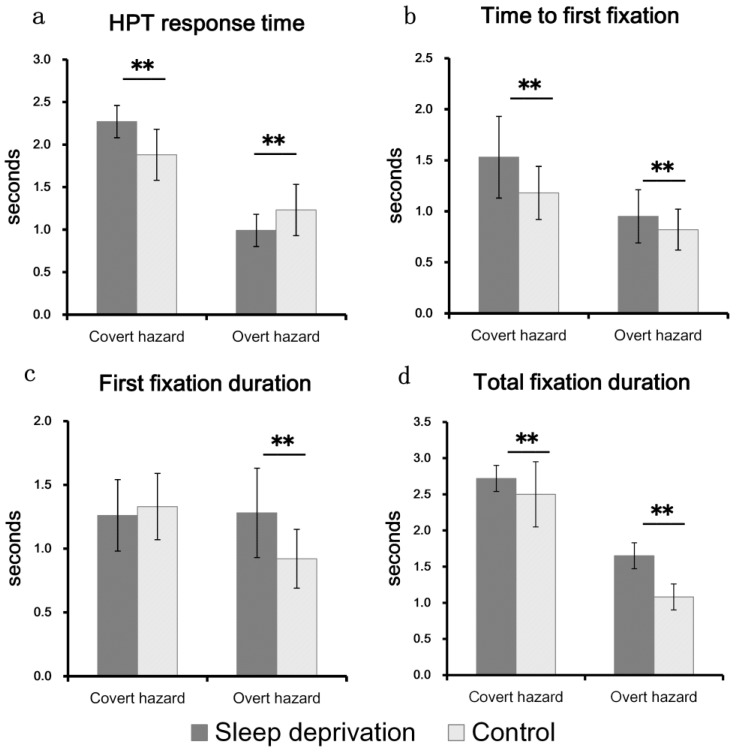
Dependent variables across driver groups and hazard types. Standard error bars included. ** *p* < 0.01.

The interaction between hazard types and sleep deprivation was significant, *F*(1, 58) = 69.52, *p* < 0.01, *ηp*^2^ = 0.545. Sample effects analysis (see Figure 3a) showed that sleep-deprived participants responded slower to covert hazards, *F*(1, 58) = 36.60, *p* < 0.001, *ηp*^2^ = 0.387, but faster to overt hazards than control drivers, *F*(1, 58) = 30.82, *p* < 0.001, *ηp*^2^ = 0.347.

#### 3.2.2. Time to First Fixation

For the time to first fixation, a significant main effect of hazard type was found, *F*(1, 58) = 94.78, *p* < 0.001, *ηp*^2^ = 0.620, indicating that participants spent a longer time obtaining the first fixation on covert hazards than overt hazards. A significant main effect of the driver group was also found, *F*(1, 58) = 17.75, *p* < 0.001, *ηp*^2^ = 0.234, indicating that sleep-deprived taxi drivers took significantly longer to first fixate on the hazard than the control group after the onset of the hazard.

The interaction between hazard types and sleep deprivation was significant, *F*(1, 58) = 5.03, *p* < 0.05, *ηp*^2^ = 0.080. Sample effects analysis (see Figure 3b) showed that the time to first fixation of sleep-deprived participants to covert hazards was significantly longer than that of the control group, *F*(1, 58) = 15.93, *p* < 0.001, *ηp*^2^ = 0.216, and also longer for overt hazards, *F*(1, 58) = 4.84, *p* < 0.05, *ηp*^2^ = 0.077.

#### 3.2.3. First Fixation Duration

For the first fixation duration, a significant main effect of hazard type was found, *F*(1, 58) = 21.94, *p* < 0.001, *ηp*^2^ = 0.274, with a longer first fixation duration for covert hazards than overt hazards. A significant main effect of the driver group was also found, *F*(1, 58) = 5.13, *p* < 0.001, *ηp*^2^ = 0.081, indicating that sleep-deprived drivers fixated significantly longer on the hazards.

The interaction between hazard types and sleep deprivation was significant, *F*(1, 58) = 26.33, *p* < 0.001, *ηp*^2^ = 0.312. Sample effects analysis (see Figure 3c) showed no significant difference in the first fixation duration for covert hazards, *F*(1, 58) = 1.20, *p* > 0.05, *ηp*^2^ = 0.020, but for overt hazards, sleep-deprived drivers fixated on them for a significantly longer time, *F*(1, 58) = 20.57, *p* < 0.001, *ηp*^2^ = 0.262.

#### 3.2.4. Total Fixation Duration

For the total fixation duration, a significant main effect of hazard type was found, *F*(1, 116) = 625.41, *p* < 0.01, with a longer total fixation duration for covert hazards than overt hazards. A significant main effect of the driver group was also found, *F*(1, 116) = 63.22, *p* < 0.01, indicating that the total fixation duration of sleep-deprived drivers was significantly longer.

The interaction between hazard types and sleep deprivation was significant, *F*(1, 116) = 11.63, *p* < 0.01. Sample effects analysis (see Figure 3d) showed that the total fixation duration of sleep-deprived participants for covert hazards was significantly longer than that of the control group, *t*(116) = 3.211, *p* < 0.01, and also longer for overt hazards, *t*(116) = 8.034, *p* < 0.01.

## 4. Discussion

The current study attempted to explore and compare the characteristics and differences in response time and eye movements of sleep-deprived and controlled taxi drivers under different hazard types. The findings showed that sleep deprivation affects response time to hazards and visual search behaviors and these influences varied with hazard type.

### 4.1. Effect of Hazard Types and Sleep Deprivation on Response Time

The observed findings showed that the sleep-deprived group had a longer response time than the control group to covert hazards but had a shorter response time to overt hazards, which partially supported hypothesis 1. This result showed that whether sleep-deprived drivers responded slower to hazards depended on hazard type, though prior studies found that sleep-deprived drivers were less able to detect and identify hazards [12,14]. Watling et al. [20] found that sleep-deprived drivers are more likely to react more slowly to hazards, engage in risky driving behaviors, and experience road traffic accidents, which is likely because sleep deprivation can cause cognitive impairment [36] or lead taxi drivers to suffer from attention deficits, decreased alertness, and poor decision-making [37,38].

Regarding why sleep-deprived drivers responded faster to overt hazards, it is possible that the time to first fixation of sleep-deprived drivers was later than the control group in cases of overt hazards. Therefore, sleep-deprived drivers fixated on hazards at a later moment in time, so that the unfolding overt hazard had become more urgent at the moment it was fixated upon. This may be the reason why sleep-deprived drivers press the mouse button fast to these hazards.

### 4.2. Effect of Hazard Types and Sleep Deprivation on Eye Movement

The present study found that the sleep-deprived group had a significantly longer time to first fixation and total fixation duration than the control group, regardless of hazard type. The results replicated the findings of previous studies and partially supported hypothesis 2. The results observed in the present study showed that the longer response time of sleep-deprived drivers to hazards resulted from the fact that they took longer to fixate on hazards and they also spent more time processing hazards. Ahlstrom et al. [39] found that when driving with insufficient sleep, response speed decreased with increasing sleepiness, and the total fixation duration increased. In this study, the inflexible visual search pattern of sleep-deprived drivers might be due to the reduced cognitive resources and poorer cognitive functioning resulting from sleep deprivation [40]. Alternatively, due to the nature of covert hazards, sleep deprivation conditions might limit the availability of drivers’ attention resources and the acquisition of information related to covert hazards.

Notably, the present study found that the sleep-deprived group had a longer first fixation duration on overt hazards, while the first fixation duration of the two groups on covert hazards was similar. This result suggests that although sleep-deprived drivers spent similar amounts of time processing a covert hazard after fixating on it, the processing efficiency of the first fixation may be less than that of the control group (their subsequent response time was also longer). In line with the results of longer total fixation time on covert hazards, sleep-deprived drivers may terminate their fixation behavior too early the moment they first fixate on a hazard, hence requiring more fixations to gather more information to help confirm that the relevant stimulus might be a hazard. A possible explanation could be the higher risk tolerance of sleep-deprived drivers [41]. High-risk-tolerant drivers tend to have lower driving safety behaviors and higher accident tendencies. Sleep-deprived drivers have a higher risk tolerance and are more willing to recognize hazard signals as no-hazards.

Overall, the findings of the present study can help explain the phenomenon of why sleep-deprived drivers or drivers with insufficient sleep have a higher crash risk. On the one hand, sleep-deprived drivers took longer to search for and fixate on hazards in the traffic environment than the control group. On the other hand, after detecting a hazard, sleep-deprived drivers were slower to respond to the hazard, especially covert hazards.

## 5. Implications and Limitations

The results of the current study are of great significance to ensure the safety of taxi driving. First, sleep-deprived taxi drivers are slower to respond to hazards. To better ensure driving safety, taxi drivers should ensure that they get adequate sleep. Considering the characteristics of their long operation time and single working environment, it is possible to take a nap after a period of operation. It has been proven that stopping for a nap is effective in reducing the elevated risk of accidents associated with driving fatigue [42]. Second, the study found that sleep-deprived drivers had slower responses to covert hazards and took longer to fixate on covert hazards than the control group. Hence, hazard perception intervention training that contains more covert hazards should be carried out for drivers who are prone to crash risk, especially those with sleep deficiency caused by long working hours or poor sleep quality caused by sleep disorders. Third, many developing countries, such as China, have not included hazard perception in the theoretical part of the driving license procedure. Therefore, transportation policy-makers should consider incorporating hazard perception tests into professional driver assessments and testing.

There are some limitations of this study. First, a video-based hazard perception test was adopted in this study. Although sleep deprivation-related differences were found in response time and visual search patterns, future studies should try to explore whether these results can be found in studies conducted using a valid driving simulator or on the road. Second, the number of participants was small, which might limit the generalization of the results found in this study. Future studies should try to include more taxi drivers to further reassess the findings.

## 6. Conclusions

The current study examined the differences in hazard response time and visual search patterns of sleep-deprived and normal sleep taxi drivers. The main outcomes include that the effects of insufficient sleep on taxi drivers’ hazard response times and visual search patterns varied with hazard type. More importantly, drivers with sleep deprivation are slower in recognizing covert hazards and have less flexible visual search patterns. These findings show that the video-based hazard perception test is helpful for distinguishing taxi drivers and formulating intervention measures and training for sleep-deprived taxi drivers.

## Figures and Tables

**Table 1:** Demographic information.

Variables	Control (*n* = 30)	Sleep Deprivation (*n* = 30)
Gender (Male/Female)	25/5	22/8
Age (years)	34.73 ± 11.31	37.60 ± 10.87
Driving experience (years)	10.11 ± 10.22	11.25 ± 9.23
Driving frequency (per week)	3.57 ± 1.15	3.56 ± 1.72
Traffic violations	0.23 ± 0.43	0.67 ± 0.84
Traffic accidents	0.17 ± 0.38	0.37 ± 0.49

**Table 2:** Mean and SD for each dependent variable in two groups (*M ± SD*).

Dependent Variables	Covert Hazard		Overt Hazard	
	Sleep-Deprived	Control	Sleep-Deprived	Control
Response time(s)	2.27 ± 0.19	1.88 ± 0.30	0.99 ± 0.17	1.23 ± 0.15
Time to first fixation(s)	1.53 ± 0.40	1.18 ± 0.26	0.95 ± 0.26	0.82 ± 0.20
First fixation duration(s)	1.26 ± 0.28	1.33 ± 0.26	1.28 ± 0.35	0.92 ± 0.23
Total fixation duration(s)	2.72 ± 0.18	2.50 ± 0.45	1.65 ± 0.18	1.08 ± 0.18

## Data Availability

The datasets analyzed during the current study are available from the corresponding author on reasonable request.

## Appendix A

**Table A1:** Hazard description.

Clip No.	Hazard Types	Screenshots	Brief Description
1	overt hazard		A pedestrian walked into the driving lane from the right side of the road
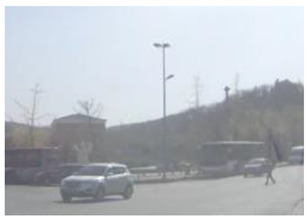
2	overt hazard		A van parked on the roadside merged into the driving lane from the right without indicating
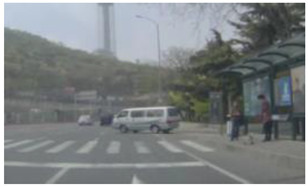
3	overt hazard		A pedestrian walked across the driving lane from the right side
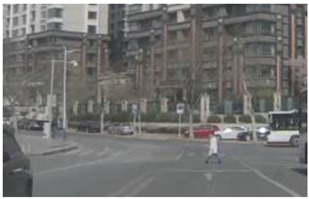
4	overt hazard		A pedestrian walked into the driving lane from the right side of the road
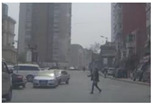
5	overt hazard		A pedestrian walked into the driving lane from the left side of the road
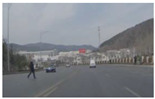
6	overt hazard		In front of the camera car, a pedestrian in the driving lane walked toward the camera car
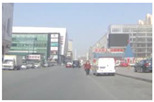
7	overt hazard		In front of the camera car, a black car going in the opposite direction pulled into the left lane
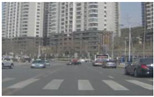
8	overt hazard		A motorcyclist was driving in the same lane in front of the camera car
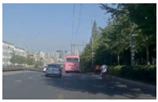
9	overt hazard		A pedestrian standing in the center of the driving lane was in front of the camera car
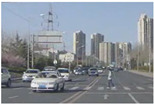
10	covert hazard		A pedestrian behind the white car, which was going in the opposite direction, walked into the driving lane from the left side
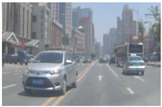
11	covert hazard		Under the overpass, a bus in the reverse lane entering the main lane from the left side was obscured by parked cars
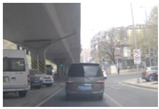
12	covert hazard		A large red van in the adjacent lane occluded a pedestrian walking across the road from the same side
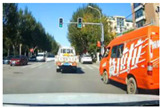
13	covert hazard		A vehicle parked on the right side of the road blocked a pedestrian going across the road
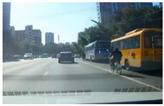
14	covert hazard		A car going in the opposite direction blocked a pedestrian crossing the road from the left side
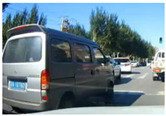
15	covert hazard		A road maintenance crew was taking a road survey but the crew was obscured by the traffic ahead
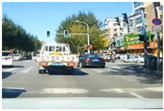
16	covert hazard		The motorcyclist entering the lane in the same direction from the other side of the road was obscured by the vehicle in front of the camera car
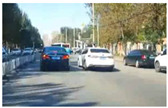
17	covert hazard		A car stuck in a traffic jam in the adjacent lane was trying to merge into the driving lane and was blocked by cars behind it
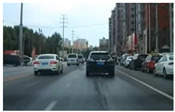
18	covert hazard		At the intersection ahead, the car going in the opposite direction on the left turned right but the camera car was blocked by a van traveling in the same direction near the lane
